# A Motion Detection Algorithm Using Local Phase Information

**DOI:** 10.1155/2016/7915245

**Published:** 2016-01-10

**Authors:** Aurel A. Lazar, Nikul H. Ukani, Yiyin Zhou

**Affiliations:** Department of Electrical Engineering, Columbia University, New York, NY 10027, USA

## Abstract

Previous research demonstrated that* global* phase alone can be used to faithfully represent visual scenes. Here we provide a reconstruction algorithm by using only* local* phase information. We also demonstrate that local phase alone can be effectively used to detect local motion. The local phase-based motion detector is akin to models employed to detect motion in biological vision, for example, the Reichardt detector. The local phase-based motion detection algorithm introduced here consists of two building blocks. The first building block measures/evaluates the temporal change of the local phase. The temporal derivative of the local phase is shown to exhibit the structure of a second order Volterra kernel with two normalized inputs. We provide an efficient, FFT-based algorithm for implementing the change of the local phase. The second processing building block implements the detector; it compares the maximum of the Radon transform of the local phase derivative with a chosen threshold. We demonstrate examples of applying the local phase-based motion detection algorithm on several video sequences. We also show how the locally detected motion can be used for segmenting moving objects in video scenes and compare our local phase-based algorithm to segmentation achieved with a widely used optic flow algorithm.

## 1. Introduction

**Figure 1:**
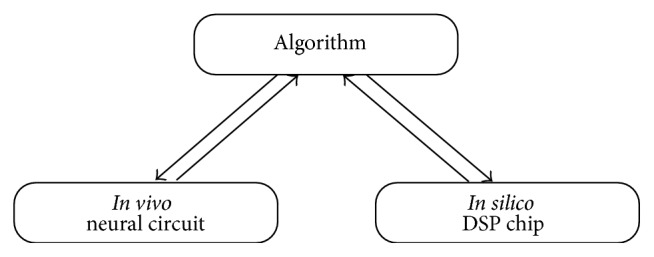
Algorithms can have different physical realizations. For example, an algorithm can be implemented by neural circuits in a biological system. Alternately, it can be implemented on a digital signal processor. Algorithms direct the implementation on the physical layer. Conversely, biological neural circuits inspire new algorithmic designs, which can, in turn, be expressed and improved upon by a realization* in silico*.

Following Marr, the design of an information processing system can be approached on multiple levels [1].  Figure 1 illustrates two levels of abstraction, namely, the algorithmic level and the physical circuit level. On the algorithmic level, one studies procedurally how the information is processed independently of the physical realization. The circuit level concerns the actual realization of the algorithm in physical hardware, for example, a biological neural circuit or silicon circuits in a digital signal processor. In this paper, we put forth a simple motion detection algorithm that is inspired by motion detection models of biological visual systems (*in vivo* neural circuit) and provide an efficient realization that can easily be implemented on commodity (*in silico*) DSP chips.

Visual motion detection is critical to the survival of animals. Many biological visual systems have evolved highly efficient/effective neural circuits to detect visual motion. Motion detection is performed in parallel with other visual coding circuits and starts already in the early stages of visual processing. In the retina of vertebrates, it is known that at least three types of Direction-Selective Ganglion Cells (DSGC) are responsible for signaling visual motion at this early stage [2]. In flies, direction-selective neurons are found in the optic lobe, 3 synapses away from the photoreceptors [3].

The small number of synapses between photoreceptors and direction-selective neurons suggests that the processing involved in motion detection is not highly complex but still very effective. In addition, the biological motion detection circuits are organized in a highly parallel way to enable fast, concurrent computation of motion. It is also interesting to note that the early stages of motion detection are carried out largely in the absence of spiking neurons, indicating that initial stages of motion detection are preferably performed in the “analog” domain. Taking advantage of continuous time processing may be critical for quickly processing motion since motion intrinsically elicits fast and large changes in the intensity levels, that is, large amounts of data under stringent time constraints.

Modern, computer-based motion detection algorithms often employ optic flow techniques to estimate spatial changes in consecutive image frames [4, 5]. Although, often time, optic flow estimation algorithms produce accurate results, the computational demand to perform many of these algorithms is too high for real-time implementation.

Several models for biological motion detection are available and their architecture is quite simple [6]. The Reichardt motion detector [7] was thought to be the underlying model for motion detection in insects [8]. The model is based on a correlation method to extract motion induced by spatiotemporal information patterns of light intensity. Therefore, it relies on a correlation/multiplication operation. A second model is the motion energy detector [9]. It uses spatiotemporal separable filters and a squaring nonlinearity to compute motion energy and it was shown to be equivalent to the Reichardt motion detector. Earlier work in the rabbit retina was the foundation to the Barlow-Levick model [10] of motion detection. The model relies on inhibition to compensate motion in the null direction.

In this paper, we provide an alternative motion detection algorithm based on local phase information of the visual scene. Similar to mechanisms in other biological models, it operates in continuous time and in parallel. Moreover, the motion detection algorithm we propose can be efficiently implemented on parallel hardware. This is, again, similar to the properties of biological motion detection systems. Rather than focusing on velocity of motion, we focus on localization, that is, where the motion occurs in the visual field as well as the direction of motion.

It has been shown that images can be represented by their global phase alone [11]. Here we provide a reconstruction algorithm of visual scenes by only using* local* phase information, thereby demonstrating the spectrum of the representational capability of phase information.

The Fourier shift property clearly suggests the relationship between the global shift of an image and the global phase shift in the frequency domain. We elevate this relationship by computing the change of local phase to indicate motion that appears locally in the visual scene. The local phases are computed using window functions that tile the visual field with overlapping segments, making it amenable for a highly parallel implementation. In addition, we propose a Radon-transform-based motion detection index on the change of local phases for the readout of the relation between local phases and motion.

Interestingly, phase information has been largely ignored in the field of linear signal processing and for good reason. Phase-based processing is intrinsically non-linear. Recent researches, however, showed that phase information can be smartly employed in speech processing [12] and visual processing [13]. For example, spatial phase in an image is indicative of local features such as edges when considering phase congruency [14]. The role of spatial phase in computational and biological vision, emergence of visual illusions, and pattern recognition is discussed in [15]. Together with our result for motion detection, these studies suggest that phase information has a great potential for achieving efficient visual signal processing.

This paper is organized as follows. In Section 2, we show that local phase information can be used to faithfully represent visual information in the absence of amplitude information. In Section 3, we develop a simple local phase-based algorithm to detect motion in visual scenes and provide an efficient way for its implementation. We then provide examples and applications of the proposed motion detection algorithm to motion segmentation. We also compare our results to those obtained using motion detection algorithms based on optic flow techniques in Section 4. Finally, we summarize our results in Section 5.

## 2. Representation of Visual Scenes Using Phase Information

The use of complex valued transforms is widespread, for both representing and processing images. When represented in polar coordinates, the output of a complex valued transform of a signal can be split into amplitude and phase. In this section, we define two types of phases of an image: global phase and local phase. We then argue that both types of phases can faithfully represent an image and we provide a reconstruction algorithm that recovers the image from local phase information. This indicates that phase information alone can largely represent an image or video signal.

It will become clear in the sections that follow that the use of phase for representing of image and video information leads to efficient ways to implement certain types of image/video processing algorithms, for example, motion detection algorithms.

### 2.1. The Global Phase of Images

Recall that the Fourier transform of a real valued image *u* = *u*(*x*, *y*), (*x*, *y*) ∈ *ℝ* ^2^, is given by

(1) $$\begin{array}{c} \hat{U}\left(\;\omega_{x},\omega_{y}\;\right)=\int_{R^{2}}u\left(\;x,y\;\right)e^{-j\left(\omega_{x}x+\omega_{y}y\right)}dx\;dy \end{array}$$

with $\hat{U}(\omega_{x},\omega_{y})\in \mathbb{C}$ and (*ω*_*x*_, *ω*_*y*_) ∈ *ℝ* ^2^.

In polar coordinates, the Fourier transform of *u* can be expressed as

(2) $$\begin{array}{c} \hat{U}\left(\;\omega_{x},\omega_{y}\;\right)=\hat{A}\left(\;\omega_{x},\omega_{y}\;\right)e^{j\hat{\phi }\left(\omega_{x},\omega_{y}\right)}, \end{array}$$

where $\hat{A}(\omega_{x},\omega_{y})\in \mathbb{R}$ is the amplitude and $\hat{\phi }(\omega_{x},\omega_{y})$ is the phase of the Fourier transform of *u*.

**Definition 1 .**

The amplitude of the Fourier transform of an image *u* = *u*(*x*, *y*), (*x*, *y*) ∈ *ℝ* ^2^, is called the global amplitude of *u*. The phase of the Fourier transform of an image *u* = *u*(*x*, *y*), (*x*, *y*) ∈ *ℝ* ^2^, is called the global phase of *u*.

It is known that the global phase of an image plays an important role in the representation of natural images [11]. A classic example is to take two images and to exchange their global phases before their reconstruction using the inverse Fourier transform. The resulting images are slightly smeared but largely reflect the information contained in the global phase.

### 2.2. The Local Phase of Images

The global phase indicates the offset of sinusoids of different frequencies contained in the entire image. However, it is not intuitive to relate the global phase to local image features such as edges and their position in an image. To study these local features, it is necessary to modify the Fourier transform such that it reflects properties of a restricted region of an image. It is natural to consider the Short-Time (-Space) Fourier Transform (STFT):

(3) Uωx,ωy,x0,y0=∫R2ux,ywx−x0,y−y0·e−jωxx−x0+ωyy−y0dx dy,

where *w* = *w*(*x*, *y*), (*x*, *y*) ∈ *ℝ* ^2^, is a real valued window function centered at (*x*_0_, *y*_0_) ∈ *ℝ* ^2^. Typical choices of window functions include the Hann window and the Gaussian window. The (effectively) finite support of the window restricts the Fourier transform to local image analysis.

Similarly to the Fourier transform, the STFT can be expressed in polar coordinates as

(4) $$\begin{array}{c} U\left(\;\omega_{x},\omega_{y},x_{0},y_{0}\;\right)=A\left(\;\omega_{x},\omega_{y},x_{0},y_{0}\;\right)e^{j\phi \left(\omega_{x},\omega_{y},x_{0},y_{0}\right)}, \end{array}$$

where *A*(*ω*_*x*_, *ω*_*y*_, *x*_0_, *y*_0_) ∈ *ℝ* is the amplitude and *ϕ*(*ω*_*x*_, *ω*_*y*_, *x*_0_, *y*_0_) is the phase of the STFT.

**Definition 2 .**

The amplitude of the STFT of an image *u* = *u*(*x*, *y*), (*x*, *y*) ∈ *ℝ* ^2^, is called the local amplitude of *u*. The phase of the STFT of an image *u* = *u*(*x*, *y*), (*x*, *y*) ∈ *ℝ* ^2^, is called the local phase of *u*.

Note that when *w* is a Gaussian window, the STFT of *u* evaluated at (*ω*_*x*_, *ω*_*y*_, *x*_0_, *y*_0_) can be equivalently viewed as the response of a complex-valued Gabor receptive field

(5) $$\begin{array}{c} h\left(\;x,y\;\right)=e^{-\left({\left(x-x_{0}\right)}^{2}+{\left(y-y_{0}\right)}^{2}\right)/2\sigma^{2}}e^{-j\left(\omega_{x}\left(x-x_{0}\right)+\omega_{y}\left(y-y_{0}\right)\right)} \end{array}$$

to *u*.

In this case, the window of the STFT clearly is given by

(6) $$\begin{array}{c} w\left(\;x,y\;\right)=e^{-\left(x^{2}+y^{2}\right)/2\sigma^{2}}. \end{array}$$

Therefore, the STFT can be realized by an ensemble of Gabor receptive fields that are common in modeling simple and complex cells (neurons) in the primary visual cortex [16].

### 2.3. Reconstruction of Images from Local Phase

Amplitude and phase can be interpreted as measurements/projections of images that are indicative of their information content. Classically, when both the global amplitude and phase are known, it is straightforward to reconstruct the image. The reconstruction calls for computing the inverse Fourier transform given the global amplitude and phase. Similarly, when using local amplitude and phase, if the sampling functions form a basis or frame in a space of images, the reconstruction is provided by the formalism of wavelet theory, for example, using Gabor wavelets [17].

Amplitude or phase represents partial information extracted from visual scenes. They are obtained via nonlinear sampling, that is, a nonlinear operation for extracting the amplitude and phase information from images. The nonlinear operation makes reconstruction from either amplitude or phase alone difficult. Earlier studies and recent development in solving quadratic constraints, however, suggest that it is possible to reconstruct images from global or local amplitude information [18, 19].

While computing the amplitude requires a second order (quadratic) nonlinearity, computing the phase calls for higher order nonlinear operators (Volterra kernels). It is possible, however, to reconstruct up to a constant scale an image from its global phase information alone without explicitly using the amplitude information. An algorithm was provided for solving this problem in the discrete signal processing domain in [11]. The algorithm smartly avoids using the inverse tangent function by reformulating the phase measurements as a set of linear equations that are easy to solve.

Using a similar argument, we demonstrate in the following that, up to a constant scale, a bandlimited signal *u* = *u*(*x*, *y*), (*x*, *y*) ∈ *ℝ* ^2^, can be reconstructed from its local phase alone. We first formulate the encoding of an image *u* by local phase, using the Gabor receptive fields as a special case. It is straightforward then to formulate the problem with local phase computed from other types of STFTs.

Formally, we consider an image, *u* = *u*(*x*, *y*), on the domain *ℝ* ^2^, to be an element of a space of trigonometric polynomials *ℋ* of the form

(7) $$\begin{array}{c} u\left(\;x,y\;\right)=\overset{L_{x}}{\underset{l_{x}=-L_{x}}{\sum }}\;\overset{L_{y}}{\underset{l_{y}=-L_{y}}{\sum }}c_{l_{x}l_{y}}e_{l_{x}l_{y}}\left(\;x,y\;\right), \end{array}$$

where

(8) elxly=exp⁡jlxΩxLxxexp⁡jlyΩyLyy,lx=−Lx,…,Lx,  ly=−Ly,…,Ly

are the set of basis functions of *ℋ*, *Ω*_*x*_, *L*_*x*_ are the bandwidth and the order, respectively, of the space in the *x* dimension, and *Ω*_*y*_, *L*_*y*_ are the bandwidth and the order, respectively, of the space in the *y* dimension.

*ℋ* is a Reproducing Kernel Hilbert Space with inner product [20, 21]

(9) $$\begin{array}{c} \langle \;u_{1},u_{2}\;\rangle =\int_{\left[0,T_{x}\right]\times \left[0,T_{y}\right]}u_{1}\left(\;x,y\;\right)\bar{u_{2}\left(x,y\right)}dx\;dy, \end{array}$$

where *T*_*x*_ = 2*πL*_*x*_/*Ω*_*x*_, *T*_*y*_ = 2*πL*_*y*_/*Ω*_*y*_ are the period of the space in the *x* and *y* dimensions, respectively.

Consider a bank of *N* Gabor receptive fields

(10) $$\begin{array}{c} h_{kl,mn}\left(\;x,y\;\right)=T_{kl}\left(\;w\left(\;x,y\;\right)e^{-j\left(\omega_{x_{m}}x+\omega_{y_{n}}y\right)}\;\right), \end{array}$$

where *𝒯*_*kl*_ is the translation operator with *𝒯*_*kl*_ *u* = *u*(*x* − *kb*_0_, *y* − *lb*_0_), *k*, *l* ∈ *ℤ*, *b*_0_ > 0, 0 ≤ *kb*_0_ ≤ *T*_*x*_, and 0 ≤ *lb*_0_ ≤ *T*_*y*_, and *ω*_*x*_*m*__ = *mω*_0_, *ω*_*y*_*n*__ = *nω*_0_, *m*, *n* ∈ *ℤ*, *ω*_0_ > 0, −*Ω*_*x*_ ≤ *mω*_0_ ≤ *Ω*_*x*_, and −*Ω*_*y*_ ≤ *nω*_0_ ≤ *Ω*_*y*_. The responses of the Gabor receptive fields to the input *u*(*x*, *y*) are given by

(11) ∫R2ux,yhkl,mnx,ydx dy=∫R2ux,y·wx−kb0,y−lb0e−jωxmx−kb0+ωyny−lb0dx dy=Akl,mnejϕkl,mn,

where *A*_*kl*,*mn*_ ≥ 0, *A*_*kl*,*mn*_ ∈ *ℝ*, is the local amplitude and *ϕ*_*kl*,*mn*_ ∈ [0,2*π*) is the local phase.

Dividing both sides of (11) by *e* ^*jϕ*_*kl*,*mn*_^, we have

(12) ∫R2ux,ywx−kb0,y−lb0·e−jωxmx−kb0+ωyny−lb0+ϕkl,mndx dy=Akl,mn.

Since *A*_*kl*,*mn*_ ∈ *ℝ*, we have

(13) ∫R2ux,ywx−kb0,y−lb0·cos⁡ωxmx−kb0+ωyny−lb0+ϕkl,mndx dy=Akl,mn,

(14) ∫R2ux,ywx−kb0,y−lb0·sin⁡ωxmx−kb0+ωyny−lb0+ϕkl,mndx dy=0.

Note that *w*(*x* − *kb*_0_, *y* − *lb*_0_)sin⁡(*ω*_*x*_*m*__(*x* − *kb*_0_) + *ω*_*y*_*n*__(*y* − *lb*_0_) + *ϕ*_*kl*,*mn*_) is a real-valued Gabor receptive field with a preferred phase at *ϕ*_*kl*,*mn*_ + *π*/2 − *ω*_*x*_*m*__ *kb*_0_ − *ω*_*y*_*n*__ *lb*_0_.

**Remark 3 .**

Assuming that the local phase information *ϕ*_*kl*,*mn*_ is obtained via measurements, that is, filtering the image *u* with pairs of Gabor receptive fields (10), the set of linear equations (14) has a simple interpretation: the image *u* is orthogonal to the space spanned by the functions

(15) wx−kb0,y−lb0·sin⁡ωxmx−kb0+ωyny−lb0+ϕkl,mn,

where (*k*, *l*, *m*, *n*) ∈ *ℑ* with *ℑ* = {(*k*, *l*, *m*, *n*) ∈ *ℤ* ^4^∣0 ≤ *kb*_0_ ≤ *T*_*x*_, 0 ≤ *lb*_0_ ≤ *T*_*y*_, −*Ω*_*x*_ ≤ *mω*_0_ ≤ *Ω*_*x*_, −*Ω*_*y*_ ≤ *nω*_0_ ≤ *Ω*_*y*_}.

We are now in the position to provide a reconstruction algorithm of the image from phase *ϕ*_*kl*,*mn*_, (*k*, *l*, *m*, *n*) ∈ *ℑ*.

**Lemma 4 .**

*u* can be reconstructed from *ϕ*_*kl*,*mn*_, (*k*, *l*, *m*, *n*) ∈ *ℑ* as

(16) $$\begin{array}{c} u\left(\;x,y\;\right)=\overset{L_{x}}{\underset{l_{x}=-L_{x}}{\sum }}\;\overset{L_{y}}{\underset{l_{y}=-L_{y}}{\sum }}c_{l_{x}l_{y}}e_{l_{x}l_{y}}\left(\;x,y\;\right), \end{array}$$

with

(17) $$\begin{array}{c} \Phi c=0, \end{array}$$

where Φ is a matrix whose *p*th row and *q*th column entry are

(18) Φpq=∫R2wx−kb0,y−lb0·sin⁡ωxmx−kb0+ωyny−lb0+ϕkl,mn·elxlyx,ydx dy.

Here *p* traverses the set *ℑ*, and *q* = (2*L*_*y*_ + 1)(*l*_*x*_ + *L*_*x*_)+(*l*_*y*_ + *L*_*y*_ + 1). **c** is a vector of the form

(19) c=c−Lx,−Ly,c−Lx,−Ly+1,…,c−Lx,Ly,c−Lx+1,−Ly,c−Lx+1,−Ly+1,…,c−Lx+1,Ly,…,cLx,−Ly,cLx,−Ly+1,…,cLx,LyT

that belongs to the null space of Φ. A necessary condition for perfect reconstruction of *u*, up to a constant scale, is that *N* ≥ (2*L*_*x*_ + 1)(2*L*_*y*_ + 1) − 1, where *N* is the number of phase measurements.

**Proof**

Substituting (7) into (14), we obtain

(20) ∑lx=−LxLx ∑ly=−LyLyclxly∫R2wx−kb0,y−lb0·sin⁡ωxmx−kb0+ωyny−lb0+ϕkl,mn·elxlyx,ydx dy=0,

for all *k*, *l*, *m*, *n* ∈ *ℤ*. Therefore, we have

(21) $$\begin{array}{c} \Phi c=0, \end{array}$$

inferring that **c** is in the null space of Φ.

If *N* < (2*L*_*x*_ + 1)(2*L*_*y*_ + 1) − 1, it follows from the rank-nullity theorem that dim⁡(null(Φ)) > 1 leading to multiple linearly independent solutions to Φ**c** = 0.

**Example 5 .**

**Figure 2:**
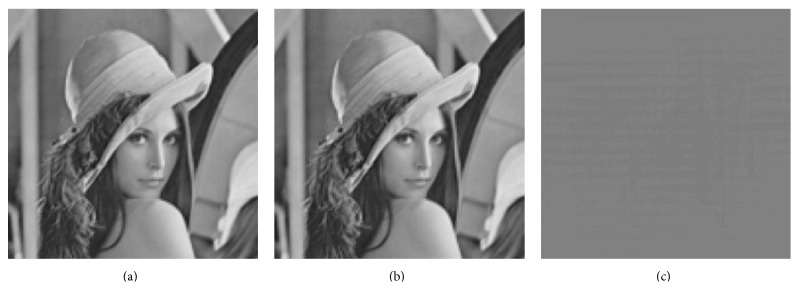
An example of reconstruction of image from local phase information. (a) Original image, the dimension of the space is 16,129. (b) Reconstructed image from 20,102 phase measurements, scaled to match the original. (c) Error.

In Figure 2 an example of reconstruction of an image is shown using only local phase information. The reconstructed signal was scaled to match the original signal. The SNR of the reconstruction is 44.48 [dB]. An alternative way to obtain a unique reconstruction is to include an additional measurement, for example, the mean value of the signal ∫_*ℝ*^2^_ *u*(*x*, *y*)*dx* *dy* to the system of linear equations (14).

## 3. Visual Motion Detection from Phase Information

In this section we consider visual fields that change as a function of time. For notational simplicity *u* will denote here the space-time intensity of the visual field.

### 3.1. The Global Phase Equation for Translational Motion

Let *u* = *u*(*x*, *y*, *t*), (*x*, *y*) ∈ *ℝ* ^2^, *t* ∈ *ℝ*, be a visual stimulus. If the visual stimulus is a pure translation of the signal at *u*(*x*, *y*, 0), that is,

(22) $$\begin{array}{c} u\left(\;x,y,t\;\right)=u\left(\;x-s_{x}\left(\;t\;\right),y-s_{y}\left(\;t\;\right),0\;\right), \end{array}$$

where

(23) sxt=∫0tvxsds,syt=∫0tvysds

are the total length of translation at time *t* in each dimension and *v*_*x*_(*t*) and *v*_*y*_(*t*) are the corresponding instantaneous velocity components, then the only difference between *u*(*x*, *y*, *t*) and *u*(*x*, *y*, 0) in the Fourier domain is captured by their global phase. More formally, consider the following.

**Lemma 6 .**

The change (derivative) of the global phase is given by

(24) dϕ^ωx,ωy,tdt−ωxvxt−ωyvyt=−ωx,ωyvxt,vytT,

where, by abuse of notation, $\hat{\phi }\left(\omega_{x},\omega_{y},t\right)$ denotes the global phase of *u*(*x*, *y*, *t*) and $\hat{\phi }\left(\omega_{x},\omega_{y},0\right)$ is the initial condition.

**Proof**

If (*ℱu*(·, ·, 0))  (*ω*_*x*_, *ω*_*y*_) is the 2D (spatial) Fourier transform of *u* = *u*(*x*, *y*, 0), (*x*, *y*) ∈ *ℝ* ^2^, by the Fourier shift theorem, we have

(25) Fu·,·,tωx,ωy=Fu·,·,0ωx,ωye−jωxsxt+ωysyt.

For a certain frequency component (*ω*_*x*_, *ω*_*y*_), the change of its global phase over time amounts to

(26) dϕ^ωx,ωy,tdt−ωxdsxtdt−ωydsytdt=−ωxvxt−ωyvyt=−ωx,ωyvxt,vytT.

Therefore, in the simple case where the entire visual field is shifting, the derivative of the phase of Fourier components indicates motion, and it can be obtained by the inner product between the component frequency and the velocity vector.

### 3.2. The Change of Local Phase

#### 3.2.1. The Local Phase Equation for Translational Motion

The analysis in Section 3.1 applies to* global motion*. This type of motion occurs most frequently when the imaging device, either an eye or a camera, moves. Visual motion in the natural environment, however, is more diverse across the screen since it is, often time, produced by multiple moving objects. The objects can be small and the motion more localized in the visual field.

Taking the global phase of *u* will not simply reveal where motion of independent objects takes place or their direction/velocity of motion. The ease of interpretation of motion by using the Fourier transform, however, motivates us to reuse the same concept in detecting local motion. This can be achieved by restricting the domain of the visual field where the Fourier transform is applied.

To be able to detect local motion, we consider the local phase of *u*(*x*, *y*, *t*) by taking the STFT with window function *w*(*x*, *y*). Note that, the STFT and its ubiquitous implementation in DSP chips can be extensively used in any dimension. For simplicity and without loss of generality, we consider the window to be centered at (0,0). The STFT is given by

(27) ∫R2ux,y,twx,ye−jωxx+ωyydx dy=A00ωx,ωy,tejϕ00ωx,ωy,t,

where, by abuse of notation, *A*_00_(*ω*_*x*_, *ω*_*y*_, *t*) is the amplitude and *ϕ*_00_(*ω*_*x*_, *ω*_*y*_, *t*) the local phase.

Before we move on to the mathematical analysis, we can intuitively explain the relation between the change in local phase and visual motion taking place across the window support. First, if the stimulus undergoes a uniform change of intensity or it changes proportionally over time due to lighting conditions, for example, the local phase does not change since the phase is invariant with respect to intensity scaling. Therefore, the local phase does not change for such nonmotion stimuli. Second, a rigid edge moving across the window support will induce a phase change.

For a strictly translational signal within the window support (footprint), for example,

(28) ux,y,t=ux−sxt,y−syt,0,forx,y∈supp⁡wx,y,

where *s*_*x*_(*t*) and *s*_*y*_(*t*) are as defined in (23), we have the following result.

**Lemma 7 .**

Consider the following:

(29) dϕ00dtωx,ωy,t=−dsxtdtωx−dsytdtωy+v00ωx,ωy,t,

where, by abuse of notation, *ϕ*_00_(*ω*_*x*_, *ω*_*y*_, *t*) is the local phase of *u*(*x*, *y*, *t*) and *ϕ*_00_(*ω*_*x*_, *ω*_*y*_, 0) is the initial condition. The functional form of the term *𝔳*_00_ = *𝔳*_00_(*ω*_*x*_, *ω*_*y*_, *t*) is provided in the Appendix.

**Proof**

The derivation of (29) and the functional form of *𝔳*_00_ are given in the Appendix.

**Remark 8 .**

**Figure 3:**
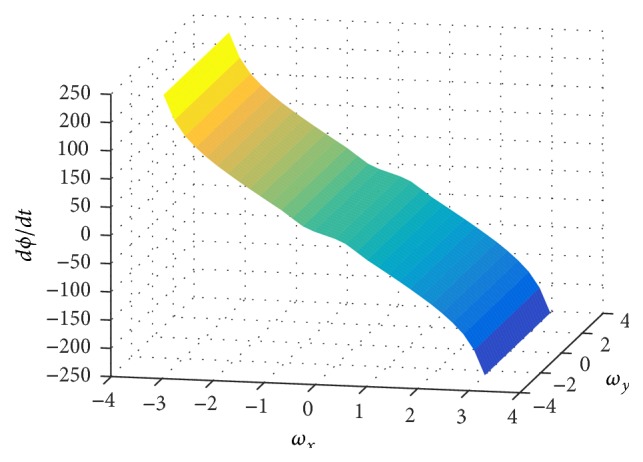
Derivative of the local phase for an ON edge moving in the positive *x* direction.

We notice that the derivative of the local phase has similar structure to that of the global phase, but for the added term *𝔳*_00_. Through simulations, we observed that the first two terms in (29) dominate over the last term for an ON or OFF moving edge [3]. For example, Figure 3 shows the derivative of the local phase given in (29) for an ON edge moving with velocity (40,0) pixels/sec.

**Remark 9 .**

Note that *ϕ*_00_(*ω*_*x*_, *ω*_*y*_, *t*) may not be differentiable even if *u*(*x*, *y*, *t*) is differentiable, particularly when *A*_00_(*ω*_*x*_, *ω*_*y*_, *t*) = 0. For example, the spatial phase can jump from a positive value to zero when *A*_00_(*ω*_*x*_, *ω*_*y*_, *t*) diminishes. This also suggests that the instantaneous local spatial phase is less informative about a region of a visual scene whenever *A*_00_(*ω*_*x*_, *ω*_*y*_, *t*) is close to zero. Nevertheless, the time derivative of the local phase can be approximated by applying a high-pass filter to *ϕ*_00_(*ω*_*x*_, *ω*_*y*_, *t*).

#### 3.2.2. The Block Structure for Computing the Local Phase

We construct Gaussian windows along the *x*, *y* dimensions. The Gaussian windows are defined as

(30) $$\begin{array}{c} \left(\;T_{kl}w\;\right)\left(\;x,y\;\right)=e^{-\left({\left(x-x_{k}\right)}^{2}+{\left(x-y_{l}\right)}^{2}\right)/2\sigma^{2}}, \end{array}$$

where *x*_*k*_ = *kb*_0_, *y*_*l*_ = *lb*_0_, in which *b*_0_ ∈ *ℤ* ^+^ is the distance between two neighboring windows and 1 ≤ *kb*_0_ ≤ *P*_*x*_, 1 ≤ *lb*_0_ ≤ *P*_*y*_, where *P*_*x*_, *P*_*y*_ ∈ *ℤ* ^+^ are the number of pixels of the screen in *x* and *y* directions, respectively.

We then take the 2D Fourier transform of the windowed video signal *u*(*x*, *y*, *t*)(*𝒯*_*kl*_ *w*)(*x*, *y*) and write in polar form

(31) ∫R2ux,y,tTklwx,ye−jωxx−xk+ωyy−yldx dy=Aklωx,ωy,tejϕklωx,ωy,t.

The above integral can be very efficiently evaluated using the 2D FFT in discrete domain defined on *M* × *M* blocks approximating the footprint of the Gaussian windows. For example, the standard deviation of the Gaussian windows we use in the examples in Section 4 is 4 pixels. A block of 32 × 32 pixels (*M* = 32) is sufficient to cover the effective support (or footprint) of the Gaussian window. At the same time, the size of the block is a power of 2, which is most suitable for FFT-based computation. The processing of each block (*k*, *l*) is independent of all other blocks; thereby, parallelism is readily achieved.

Note that the size of the window is informed by the size of the objects one is interested in locating. Measurements of the local phase using smaller window functions are less robust to noise. Larger windows would enhance object motion detection if the object size is comparable to the window size. However, there would be an increased likelihood of independent movement of multiple objects within the same window, which is not modeled here and thereby may not be robustly detected.

Therefore, for each block (*k*, *l*), we obtain *M* ^2^ measurements of the phase *ϕ*_*kl*_(*ω*_*x*_*m*__, *ω*_*y*_*m*__, *t*) at every time instant *t*, with (*ω*_*x*_*m*__, *ω*_*y*_*m*__) ∈ *𝔻* ^2^, where

(32) D2=ωxm=mω0, ωyn=nω0,  m,n=−M/2,−M/2+1,…,M/2−1,

with *ω*_0_ = 2*π*/*M*. We then compute the temporal derivative of the phase, that is, (*dϕ*_*kl*_/*dt*)(*ω*_*x*_, *ω*_*y*_, *t*) for (*ω*_*x*_, *ω*_*y*_) ∈ *𝔻* ^2^.

**Figure 4:**
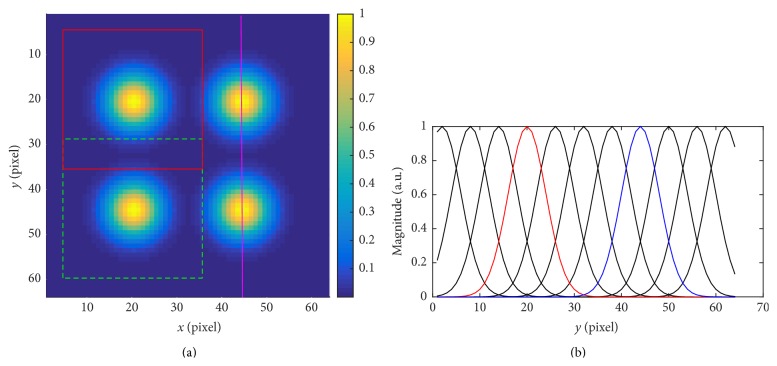
Example of block structure. (a) Four yellow disks show 4 Gaussian window functions with translation parameter *k* = 3, *l* = 3 (top-left), *k* = 3, *l* = 7 (bottom-left), *k* = 7, *l* = 3 (top-right), and *k* = 7, *l* = 7 (bottom-right). The red solid square shows a 32 × 32-pixel block approximating the Gaussian window with *k* = 3, *l* = 3, and the green dashed square shows a 32 × 32-pixel block approximating the Gaussian window with *k* = 3, *l* = 7. (b) Cross section of all 11 Gaussian windows with translation parameters *k* = 7, *l* ∈ [0,10]. The cross section is taken as indicated by the magenta line in (a), and the red and blue curve correspond to cross sections of the two Gaussian windows shown in (a) centered on the magenta line.

We further illustrate an example of the block structure in Figure 4.  Figure 4(a) shows an example of an image of 64 × 64 pixels. Four Gaussian windows are shown each with a standard deviation of 4 pixels. The distance between the centers of two neighboring Gaussian windows is 6 pixels. The red solid square shows a 32 × 32-pixel block with *k* = 3, *l* = 3, which encloses effective support of the Gaussian window on top-left (*k* = 0, *l* = 0 is the block with Gaussian window centered at pixel (1,1)). The green dashed square shows another 32 × 32-pixel block with *k* = 3, *l* = 7. The two Gaussian windows on the right are associated with the blocks *k* = 7, *l* = 3 and *k* = 7, *l* = 7, respectively. Cross section of all Gaussian windows with *k* = 7, *l* ∈ [0,10], that is, those centered on the magenta line, are shown in Figure 4(b). The red and blue curve in Figure 4(b) correspond to the two Gaussian windows shown in Figure 4(a). Figure 4(b) also suggests that some of the Gaussian windows are cut off on the boundaries. This is, however, equivalent to assuming that the pixel values outside the boundary are always zero, and it will not significantly affect motion detection based on the change of local phase.

Since the phase, and thereby the phase change, is noisier when the local amplitude is low, an additional denoising step can be employed to discount the measurements of (*dϕ*_*kl*_/*dt*)(*ω*_*x*_, *ω*_*y*_, *t*) for low amplitude values *A*_*kl*_(*ω*_*x*_, *ω*_*y*_, *t*). The denoising is given by

(33) dϕkldtωx,ωy,t·Aklωx,ωy,t1/M2∑ωx,ωy∈D2Aklωx,ωy,t+ϵ,

where *ϵ* > 0 is a constant, and (*ω*_*x*_, *ω*_*y*_) ∈ *𝔻* ^2^.

### 3.3. The Phase-Based Detector

We propose here a block FFT based algorithm to detect motion using phase information. Such an algorithm is, due to its simplicity and parallelism, highly suitable for an* in silico* implementation.

#### 3.3.1. Radon Transform on the Change of Phases

We exploit the approximately linear structure of the phase derivative for blocks exhibiting motion by computing the Radon transform of (*dϕ*_*kl*_/*dt*)(*ω*_*x*_, *ω*_*y*_, *t*) over a circular bounded domain *C* = {(*ω*_*x*_, *ω*_*y*_)∣(*ω*_*x*_, *ω*_*y*_) ∈ *𝔻* ^2^,  *ω*_*x*_^2^ + *ω*_*y*_^2^ < *π* ^2^}.

The Radon transform of the change of phase in the domain *C* is given by

(34) Rdϕkldtρ,θ,t=∫Rdϕkldtρ·cos⁡θ−s·sin⁡θ,ρ·sin⁡θ+s·cos⁡θ,t1Cρ·cos⁡θ−s·sin⁡θ,ρ·sin⁡θ+s·cos⁡θds,

where

(35) $$\begin{array}{c} 1_{C}\left(\;\omega_{x},\omega_{y}\;\right)=\left\{\begin{array}{cc} 1 & \text{if }\left(\omega_{x},\omega_{y}\right)\in C\\ 0 & otherwise. \end{array}\right. \end{array}$$

The Radon transform (*ℛ*(*dϕ*_*kl*_/*dt*))(*ρ*, *θ*, *t*) evaluated at a particular point (*ρ*_0_, *θ*_0_, *t*_0_) is essentially an integral of (*dϕ*_*kl*_/*dt*)(*ω*_*x*_, *ω*_*y*_, *t*_0_) along a line oriented at angle *π*/2 + *θ*_0_ with the *ω*_*x*_ axis and at distance |*ρ*_0_| along the (cos⁡(*θ*_0_), sin⁡(*θ*_0_)) direction from (0,0).

If, for a particular *k* and *l*, we have (*dϕ*_*kl*_/*dt*)(*ω*_*x*_, *ω*_*y*_, *t*) = −*v*_*x*_(*t*)*ω*_*x*_ − *v*_*y*_(*t*)*ω*_*y*_, we have

(36) Rdϕkl/dtρ,θ,tcρ,θ=ρ−vxtcos⁡θ−vytsin⁡θ,

where

(37) cρ,θ=∫R1Cρ·cos⁡θ−s·sin⁡θ,ρ·sin⁡θ+s·cos⁡θds.

*𝔠*(*ρ*, *θ*) is a correction term due to different length of line integrals for different values of (*ρ*, *θ*) in the bounded domain *C*.

After computing the Radon transform of (*dϕ*_*kl*_/*dt*)(*ω*_*x*_, *ω*_*y*_, *t*) for every block (*k*, *l*) at time *t*_0_, we compute the Phase Motion Indicator (PMI), defined as

(38) $$\begin{array}{c} {PMI}_{kl}=\underset{\theta \in \left[0,\pi \right)}{\max}\underset{\rho }{\sum }\left|\;\frac{\left(R\left(d\phi_{kl}/dt\right)\right)\left(\rho ,\theta ,t_{0}\right)}{c\left(\rho ,\theta \right)}\;\right|. \end{array}$$

If the PMI_*kl*_ is larger than a chosen threshold, motion is deemed to occur in block (*k*, *l*) at time *t*_0_.

Using the Radon transform makes it easier to separate rigid motion from noise. Since the phase is quite sensitive to noise, particularly when the amplitude is very small, the change of phase under noise may have comparable magnitude to that due to motion as mentioned earlier. The change of phase under noise, however, does not possess the structure suggested by (29) in the (*ω*_*x*_, *ω*_*y*_) domain. Instead, it appears to be more randomly distributed. Consequently, the PMI value is comparatively small for these blocks (see also Section 3.3.3).

Moreover, the direction of motion, for block (*k*, *l*) where motion is detected, can be easily computed as

(39) θ^kl=πsign⁡∑ρ>0Rdϕkl/dtρ,αkl,t0/cρ,αkl+12+αkl,

where

(40) $$\begin{array}{c} \alpha_{kl}=\underset{\theta \in \left[0,\pi \right)}{\mathrm{argmax}}\underset{\rho }{\sum }\left|\;\frac{\left(R\left(d\phi_{kl}/dt\right)\right)\left(\rho ,\theta ,t_{0}\right)}{c\left(\rho ,\theta \right)}\;\right|. \end{array}$$

This follows from (36).

#### 3.3.2. The Phase-Based Motion Detection Algorithm

**Figure 5:**
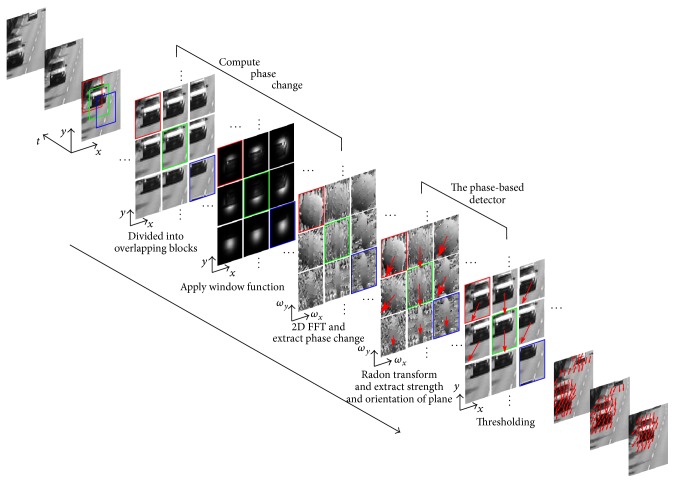
Schematic diagram of proposed phase-based motion detection algorithm.

We formally summarize the above analysis as Algorithm 1.  Figure 5 shows a schematic diagram of the proposed phase-based motion detection algorithm.

The algorithm is subdivided into two parts. The first part computes local phase changes and the second part is the phase-based motion detector.

In the first part, the screen is divided into overlapping blocks. For example, the red, green, and blue blocks in the plane “divide into overlapping blocks” correspond to the squares of the same color covering the video stream. A Gaussian window is then applied on each block, followed by a 2D FFT operation that is used to extract the local phase. A temporal high-pass filter is then employed to extract phase changes.

In the second part, the PMI is evaluated for each block based on the Radon transform of the local phase changes in each block. Motion is detected for blocks with PMI larger than a preset threshold, and the direction of motion is computed as in (39).

It is easy to notice that the algorithm can be highly parallelized.

#### 3.3.3. Example

**Figure 6:**
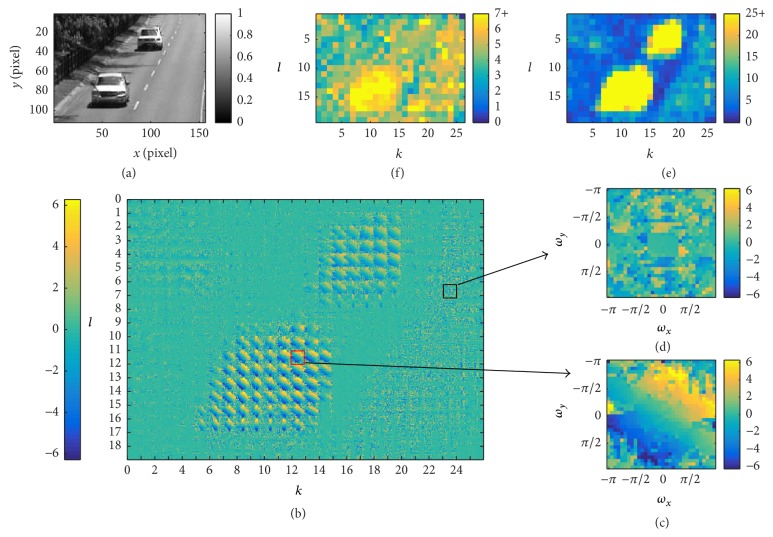
Evaluating the motion detection algorithm at time *t*_0_ (see also Supplementary Video S1). (a) A still from a 156 × 112-pixel video at time *t*_0_. (b) (*dϕ*_*kl*_/*dt*)(*ω*_*x*_, *ω*_*y*_, *t*_0_) for all 26 × 19 blocks, each of size 32 × 32. The blocks are concatenated to create a large “phase image” with their relative neighbors kept. (c) (*dϕ*_12,11_/*dt*)(*ω*_*x*_, *ω*_*y*_, *t*_0_) shown in the (*ω*_*x*_, *ω*_*y*_) ∈ *𝔻* ^2^ space. (d) (*dϕ*_23,6_/*dt*)(*ω*_*x*_, *ω*_*y*_, *t*_0_) shown in the (*ω*_*x*_, *ω*_*y*_) ∈ *𝔻* ^2^ space. (e) Phase Motion Indicator (PMI) for all blocks at *t*_0_. Each pixel (*k*, *l*) is color coded for PMI_*kl*_. (f) max_(*ω*_*x*_, *ω*_*y*_)∈*𝔻*^2^_⁡|(*dϕ*_*kl*_/*dt*)(*ω*_*x*_, *ω*_*y*_, *t*_0_)| for all blocks at *t*_0_. Each pixel (*k*, *l*) is color coded for this maximum value of block (*k*, *l*).

We provide an illustrative example in Figure 6 showing how motion is detected using Algorithm 1. The full video of this example can be found in Supplementary Video S1; see Supplementary Material available online at http://dx.doi.org/10.1155/2016/7915245. Figure 6(a) depicts a still from the “highway video” in the Change Detection 2014 dataset [22] evaluated at a particular time *t*_0_. As suggested by the algorithm, the screen in Figure 6(a) is divided into 26 × 19 overlapping blocks and the window functions are applied to each block. Local phases can then be extracted from the 2D FFT of each windowed block, and the local phase changes are obtained by temporal high-pass filtering. The phase change is shown in Figure 6(b) for all blocks, with block (12,11) enlarged in Figure 6(c) and block (23,6) enlarged in Figure 6(d) (see also the plane “2D FFT and extract phase change” in Figure 5). Note that at the time of the video frame, block (12,11) covers a part of the vehicle in motion in the front, and block (23,6) corresponds to an area of the highway pavement where no motion occurs.

Figure 6(f) depicts, for each block (*k*, *l*), the maximum phase change over all (*ω*_*x*_, *ω*_*y*_) ∈ *𝔻* ^2^; that is,

(41) $$\begin{array}{c} \underset{\left(\omega_{x},\omega_{y}\right)\in D^{2}}{\max}\left|\;\frac{d\phi_{kl}}{dt}\left(\;\omega_{x},\omega_{y},t_{0}\;\right)\;\right|. \end{array}$$

We observe from the figure that, for regions with low amplitude, such as the region depicting the road, when the normalization constant is absent, the derivative of the phase can be noisy. For these blocks the maximum of |(*dϕ*_*kl*_/*dt*)(*ω*_*x*_, *ω*_*y*_, *t*_0_)| over all (*ω*_*x*_, *ω*_*y*_) ∈ *𝔻* ^2^ is comparable to the maximum obtained for blocks that cover the vehicles in motion.

However, (29) suggests that the local phase change from multiple filter pairs centered at the same spatial position (*k*, *l*) can provide a constraint to robustly estimate motion and its direction. Given the block structure employed in the computation of the local phase, it is natural to utilize phase change information from multiple sources.

Indeed, if, for a particular block (*k*, *l*), (*dϕ*_*kl*_/*dt*)(*ω*_*x*_, *ω*_*y*_, *t*) = −*v*_*x*_(*t*)*ω*_*x*_ − *v*_*y*_(*t*)*ω*_*y*_, then it is easy to see that (*dϕ*_*kl*_/*dt*)(*ω*_*x*_, *ω*_*y*_, *t*) will be zero on the line *v*_*x*_(*t*)*ω*_*x*_ + *v*_*y*_(*t*)*ω*_*y*_ = 0 and have opposite sign on either side of this line. For example, in Figures 6(b) and 6(c), (*dϕ*_*kl*_/*dt*)(*ω*_*x*_, *ω*_*y*_, *t*_0_) clearly exhibits this property for blocks that cover a vehicle in motion. The PMI is a tool to evaluate this property.

Finally, the PMIs for all blocks are shown compactly in a heat map in Figure 6(e). The figure shows clearly that the blocks corresponding to the two moving vehicles have a high PMI value while the stationary background areas have a low PMI value, allowing one to easily detect motion by employing simple thresholding (see also the plane “Radon transform and extract strength orientation of plane” in Figure 5). In addition, the orientation of motion in each block is readily observable even by inspection in Figure 6(b) by a line separating the yellow part and blue part in each block. Further results about the direction of motion are presented in Section 4.

### 3.4. Relationship to Biological Motion Detectors

A straightforward way to implement local motion detectors is to apply a complex-valued Gabor receptive field (5) to the video signal *u*, and then take the derivative of the phase with respect to time or apply a high-pass filter on the phase to approximate the derivative.

We present here an alternate implementation without explicitly computing the phase. This will elucidate the relation between the phase-based motion detector presented in the previous section and some elementary motion detection models used in biology, such as the Reichardt motion detector [7] and motion energy detector [9].

Assuming that the local phase *ϕ*_00_(*ω*_*x*_, *ω*_*y*_, *t*) is differentiable, we have (see also the Appendix)

(42) $$\begin{array}{c} \frac{d\phi_{00}\left(\omega_{x},\omega_{y},t\right)}{dt}=\frac{\left(db\left(\omega_{x},\omega_{y},t\right)/dt\right)a\left(\omega_{x},\omega_{y},t\right)-\left(da\left(\omega_{x},\omega_{y},t\right)/dt\right)b\left(\omega_{x},\omega_{y},t\right)}{{\left[a\left(\omega_{x},\omega_{y},t\right)\right]}^{2}+{\left[b\left(\omega_{x},\omega_{y},t\right)\right]}^{2}}, \end{array}$$

where *a*(*ω*_*x*_, *ω*_*y*_, *t*) and *b*(*ω*_*x*_, *ω*_*y*_, *t*) are, respectively, the real and imaginary parts of *A*_00_(*ω*_*x*_, *ω*_*y*_, *t*)*e* ^*jϕ*_00_(*ω*_*x*_,*ω*_*y*_,*t*)^.

We notice that the denominator of (42) is the square of the local amplitude of *u*, and the numerator is of the form of a second order Volterra kernel. This suggests that the time derivative of the local phase can be viewed as a second order Volterra kernel that processes two* normalized* spatially filtered inputs *v*_1_ and *v*_2_.

**Figure 7:**
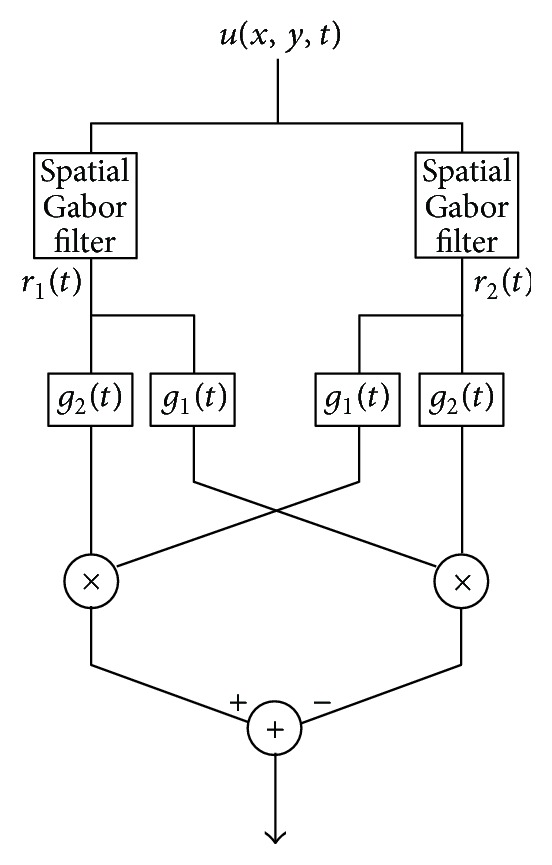
Diagram of an elaborated Reichardt motion detector. It consists of a quadrature pair of Gabor filters whose outputs *r*_1_ and *r*_2_ provide inputs to the high-pass filters *g*_1_(*t*) and the low-pass filters *g*_2_(*t*).

We consider an elaborated Reichardt motion detector as shown in Figure 7. It is equipped with a quadrature pair of Gabor filters whose outputs are *r*_1_(*t*) = *a*(*ω*_*x*_, *ω*_*y*_, *t*) and *r*_2_(*t*) = *b*(*ω*_*x*_, *ω*_*y*_, *t*), respectively, for a particular value of (*ω*_*x*_, *ω*_*y*_). The pair of Gabor filters that provide these outputs are the real and imaginary parts of *w*(*x*, *y*)*e* ^−*j*(*ω*_*x*_*x*+*ω*_*y*_*y*)^. It also consists of a temporal high-pass *g*_1_(*t*) and temporal low-pass filter *g*_2_(*t*) [9]. The output of the elaborated Reichardt detector follows the diagram in Figure 7 and can be expressed as

(43) $$\begin{array}{c} \left(\;r_{2}*g_{1}\;\right)\left(\;t\;\right)\left(\;r_{1}*g_{2}\;\right)\left(\;t\;\right)-\left(\;r_{1}*g_{1}\;\right)\left(\;t\;\right)\left(\;r_{2}*g_{2}\;\right)\left(\;t\;\right). \end{array}$$

The response can also be characterized by a second order Volterra kernel. We notice the striking similarity between (43) and the numerator of (42). In fact, the phase-based motion detector shares some properties with the Reichardt motion detector. For example, it is straightforward to see that a single phase-based motion detector is tuned to the temporal frequency of a moving sinusoidal grating.

Since the motion energy detector is formally equivalent to an elaborated Reichardt motion detector [9], the structure of the motion energy detector with divisive normalization is also similar to the phase-based motion detector.

## 4. Exploratory Results

In this section, we apply the phase-based motion detection algorithm on several video sequences and demonstrate its efficiency and effectiveness in detecting local motion. The motion detection algorithm is compared to two well-known biological motion detectors, namely, the Reichardt motion detector and the Barlow-Levick motion detector. We then show that the detected local motion can be used in motion segmentation tasks. The effectiveness of the segmentation is compared to segmentation using motion information obtained from a widely used optic flow based algorithm available in the literature [4].

### 4.1. Efficient Parallel Implementation

The algorithm was implemented in PyCUDA [23] and tested on an NVIDIA GeForce GTX TITAN GPU. All computations use single precision floating points. The processing speeds of the algorithm for several screen sizes are listed in Table 1. Clearly, the proposed phase-based motion detection algorithm has real-time capability to process video even with full High Definition screen size.

**Figure 8:**
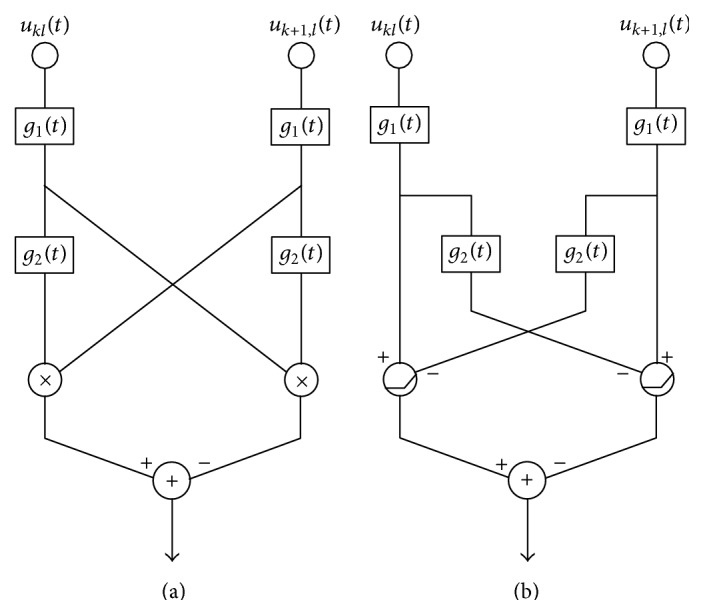
(a) A Reichardt motion detector detecting motion between the two points *u*_*kl*_ and *u*_*k*+1,*l*_ in the blurred and downsampled video. (b) A Barlow-Levick motion detector detecting motion between the two points *u*_*kl*_ and *u*_*k*+1,*l*_ in the blurred and downsampled video. *g*_1_(*t*) denotes a high-pass filter and *g*_2_(*t*) denotes a low-pass filter.

For comparison, we implemented the Reichardt motion detector and the Barlow-Levick motion detector. Their respective diagrams are shown in Figure 8. Note that we moved the high-pass filter *h*_1_(*t*) to the front of the low-pass filter *h*_2_(*t*). This configuration provides a superior performance to the one in Figure 7. For both the Reichardt and the Barlow-Levick detectors, the videos are first blurred by a Gaussian filter (with the same variance as the Gaussian window in (30) used for the phase-based motion detector) and subsampled at the center of each overlapping block in the phase-based motion detector. The subsampled video then provides inputs to two 2D arrays of the circuits shown in Figure 8, one for the horizontal direction and one for the vertical direction. The outputs of the horizontal and vertical motion circuits form an array of motion vectors that indicate the strength and direction of motion. The three tested motion detectors have the same number of outputs as a result. The processing speeds of the Reichardt motion detector and the Barlow-Levick motion detector, both implemented in PyCUDA and tested on the same GPU, are shown in Table 1.

Note that the Reichardt motion detector and the Barlow-Levick motion detector are highly efficient due to the simplicity of their algorithms. The phase-based motion detection algorithm, however, is a much more sophisticated algorithm, and yet it can be implemented in real-time using parallel computing devices.

The fast GPU implementation is based on the FFT and Matrix-Matrix multiplication. It is expected that those operations can be efficiently implemented in hardware, for example, FPGA.

### 4.2. Examples of Phased-Based Motion Detection

We applied our motion detection algorithm on video sequences of the Change Detection 2014 dataset [22] that did not exhibit camera egomotion. For these video sequences, the standard deviation of the Gaussian window functions was set to 4 pixels and the block size was chosen to be 32 × 32 pixels. Threshold and normalization parameters were kept the same with the exception of the “thermal video” (in order to deal with larger background noise levels, see below). We also tested the same video sequences using the Reichardt motion detector and the Barlow-Levick motion detector. For the Reichardt detector, the high-pass filters were chosen as first order filters with a time constant of 200 milliseconds, and the low-pass filters were chosen as first order filters with a time constant of 300 milliseconds (assuming that the frame rate is 50 frames per second). Threshold was set to 2. For the Barlow-Levick motion detector, the time constant of the first high-pass filters was set to 250 milliseconds. The low-pass filters were the same as in the Reichardt detector. Threshold was set to 2.

**Figure 9:**
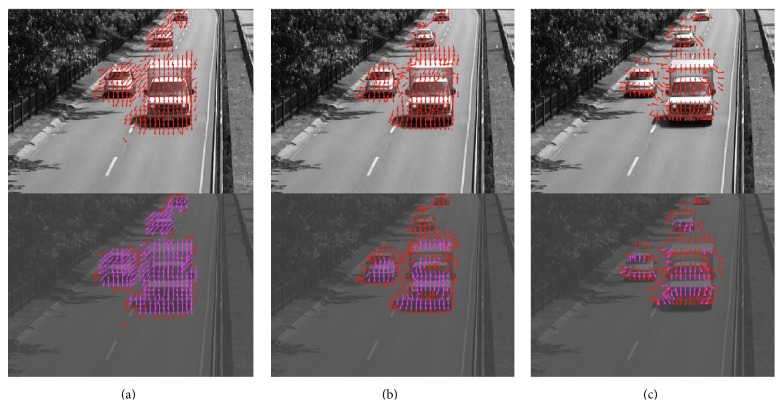
Top: motion detection of the “highway video” using the phase-based algorithm (a), the Reichardt motion detector (b), and the Barlow-Levick motion detector (c). Red arrows indicate detected motion and its direction. Bottom: the contrast of the video was artificially reduced by 5-fold and the mean was reduced to 3/5 of the original. The red arrows are duplicated from the motion detection result on the original video as in top. Blue arrows are the result of motion detection on the video with reduced contrast. If motion is detected both in the original video and in the video with reduced contrast, then the arrow is shown in magenta (as a mix of blue and red) (see Supplementary Video S2 for full video).

The first video was taken from a highway surveillance camera (“highway video”) under good illumination conditions and high contrast. The video had moderate noise, particularly on the road surface. The detected motion is shown in the top left panel of Figure 9 (see Supplementary Video S2 for full video). The phase-based motion detection algorithm captured both the moving cars and the tree leaves moving (due to the wind). In the time interval between the 9th and 10th second, the camera was slightly moved left- and right-wards within 5 frames, again possibly due to the wind. Movement due to this shift was captured by the motion detection algorithm and the algorithm was fast enough to correctly determine the direction of this movement. In this video, we already noted that this algorithm suffers from the aperture problem. For example, in front of the van where a long, horizontal edge is present, the detected motion is mostly pointing downwards. In addition to moving downwards, the edge is also moving to the left, however. This is expected since the algorithm only detects motion locally and does not take into account the overall shape of any object.

For comparison, the motion detection results for the Reichardt motion detector and the Barlow-Levick motion detector are shown in the top middle and top right panel of Figure 9 (see Supplementary Video S2 for full video). The Reichardt detector performed relatively well when vehicles are moving faster, but the direction it predicts for vehicles moving slower, for example, on the back of the image is not accurate. In addition, motion is still detected in some parts of the screen where the vehicles have just passed by. The detection result for the Barlow-Levick motion detector was poorer. In particular, the response to OFF edge movement is always the opposite to the actual movement direction.

We then squeezed the range of the screen intensity from [0,1] to [0.2,0.4], resulting in a video with a lower mean luminance and lower contrast. The motion detection results on the low-contrast video are shown in the bottom 3 panels of Figure 9 (see Supplementary Video S2 for full video). For reference, the motion detection results for the original video are shown in red arrows. Motion detected in the low-contrast video is shown in blue arrows if no motion is detected for the block in the original video. If motion is detected in both the original and the low-contrast video, the arrows are shown in magenta. Figure 9 clearly shows that while the motion detection performance is degraded for all three detectors, the phase-based motion detector performed still quite well in the low-contrast video, detecting most of the moving vehicles. The other two detectors missed many of the blocks where motion was detected in the original movie.

**Figure 10:**
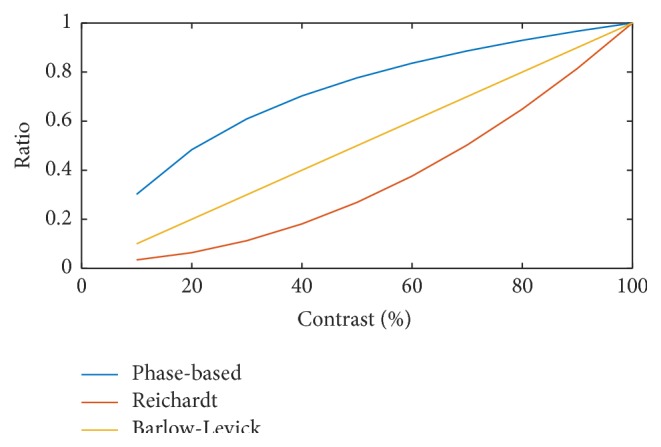
The ratio for the phase-based motion detector is defined as follows: we set the motion detected in the 100% contrast case as the baseline; we compute the ratio between the PMI index for the lower contrast video and that for the full contrast video for each block; the ratios are averaged across all the blocks where motion is detected in the full contrast video; the average is given as the ratio for the phase-based motion detector. The ratio for the Reichardt motion detector and the Barlow-Levick motion detector is similarly defined.

To quantify how well each detector works under different contrast conditions, we computed the ratio of unthresholded output values of each motion detector between lower contrast and full contrast video. For example, in the case of phase-based motion detector, we computed, for each block, the ratio between the PMI index for the lower contrast video and that for the full contrast video. The ratios are then averaged across all blocks where motion is detected in the full contrast video. This average for different contrasts is shown in Figure 10 by the blue curve. In the ideal case when the normalization constant *ϵ* is 0, the phase detector should produce invariant responses to videos with different contrast. The curve shown here is mainly due to a nonzero *ϵ*. The ratios for the Reichardt motion detector and the Barlow-Levick motion detector are computed similarly and are shown in red and yellow, respectively. It is clear that the phase-based motion detector has a superior performance across a range of contrast values. At 20% contrast, the phase-based detector still has 50% of the PMI index value for full contrast video. As expected, the response of Barlow-Levick motion detector is linear with respect to contrast, and the Reichardt motion detector has a quadratic relation to contrast. For a fixed threshold value a larger ratio equates to a more consistent performance at lower contrast.

**Figure 11:**
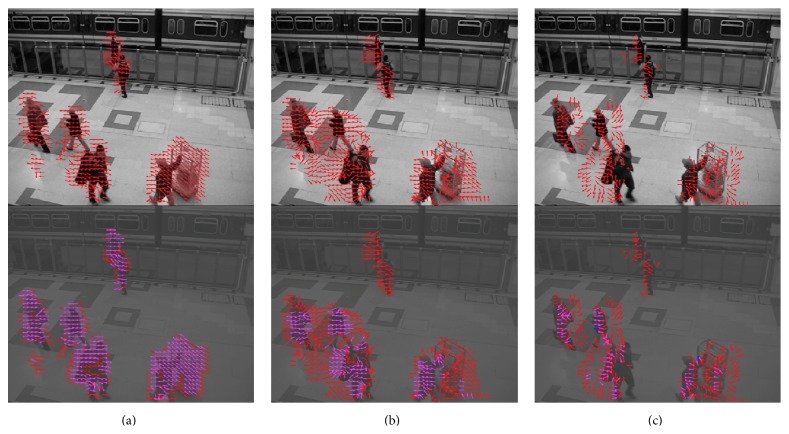
Motion detection of the “train station video” using the phase-based algorithm (a), the Reichardt motion detector (b), and the Barlow-Levick motion detector (c) (see Figure 9 for description of each panel) (see Supplementary Video S3 for full video).

The second video was captured by a surveillance camera in a train station (“train station video”). The video was under moderate room light with a low noise level. The front side of the video had high contrast; illumination on the back side was quite low. The detected motion is shown in the video of Figure 11 (see Supplementary Video S3 for full video). Movements of people were successfully captured by the motion detection algorithm.

**Figure 12:**
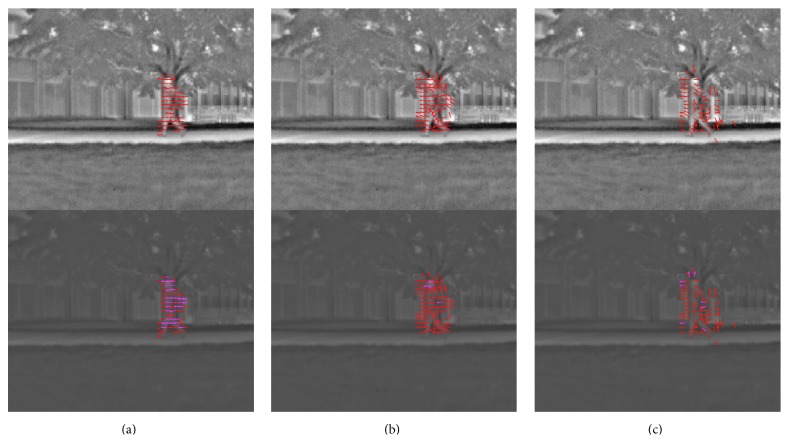
Motion detection of the “thermal video” using the phase-based algorithm (a), the Reichardt motion detector (b), and the Barlow-Levick motion detector (c) (see Figure 9 for description of each panel) (see Supplementary Video S4 for full video).

The third video was a “thermal video” with large amount of background noise (thermal video). The threshold for detecting motion was raised by 60% in order to mitigate the increased level of noise. The detected motion is shown in the video of Figure 12 (see Supplementary Video S4 for full video).

**Figure 13:**
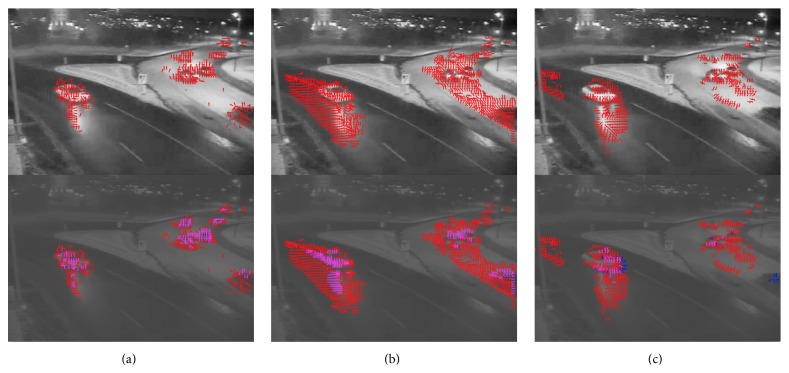
Motion detection of the “winterstreet video” using the phase-based algorithm (a), the Reichardt motion detector (b), and the Barlow-Levick motion detector (c) (see Figure 9 for description of each panel) (see Supplementary Video S5 for full video).

The last example we show here was taken from a highway surveillance camera at night (“winterstreet video”). The overall illumination on the lower-left side was low whereas illumination was moderate on the upper-right side where the road was covered by snow. The detected motions are shown in the video in Figure 13 (see Supplementary Video S5 for full video). We note that, overall, car movements were successfully detected. Car movements on the lower-left side, however, suffered from low illumination and some parts of the car were not detected well due to the trade-off employed for noise suppression.

**Figure 14:**
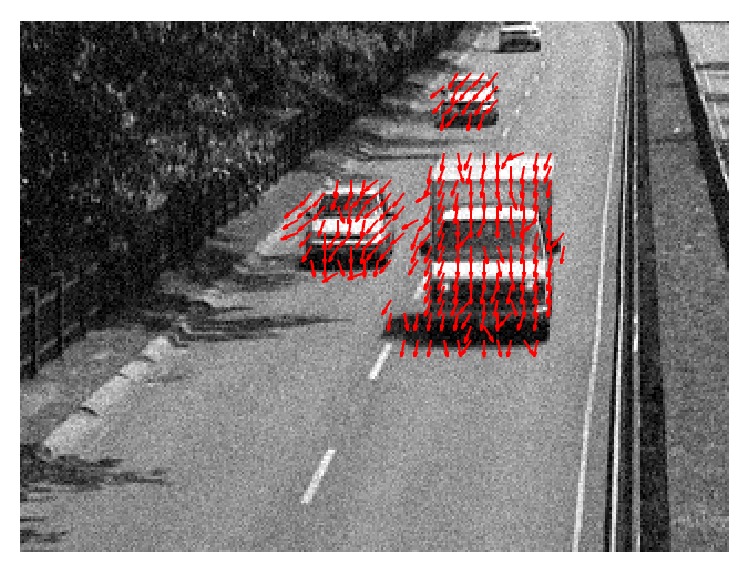
Phase-based motion detection applied on the “highway video” with added Gaussian white noise (see Supplementary Video S6 for full video).

**Figure 15:**
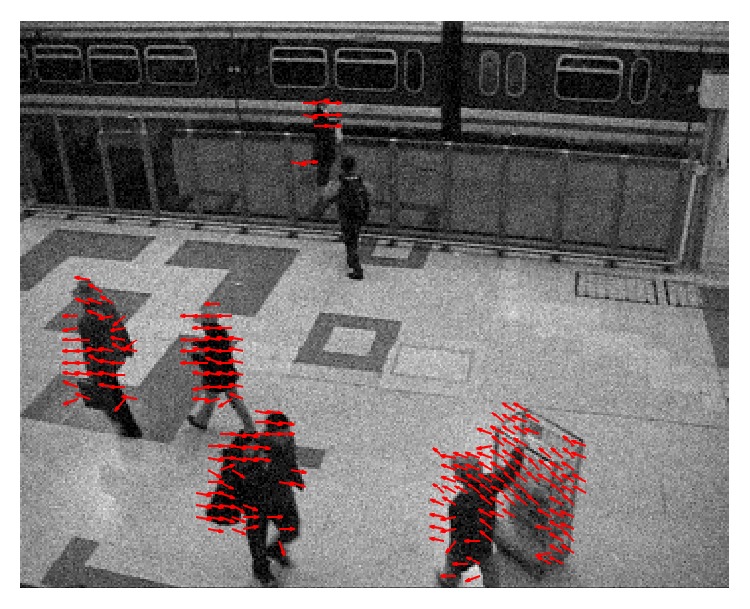
Phase-based motion detection applied on the “train station video” with added Gaussian white noise (see Supplementary Video S7 for full video).

With a higher threshold, the phase-base motion detection algorithm is able to detect motion under noisy conditions. We added to the original “highway video” and “train station video” Gaussian white noise with standard deviation 5% of the maximum luminance range. The results are shown, respectively, in Figures 14 and 15 (see Supplementary Videos S6 and S7 for full videos).

### 4.3. Examples of Motion Segmentation

We asked whether the detected motion signals in the video sequences can be useful for segmenting moving objects from the background. We applied a larger threshold to only signal motion for salient objects. The 32 × 32 blocks, however, introduce large boundaries around the moving objects. To reduce the boundary and to segment the moving object more closely to the actual object boundary, we applied the motion detection algorithm around the detected boundary with 16 × 16 blocks. If 16 × 16 blocks did not indicate motion, then the corresponding area was removed from the segmented object area.

For comparison, we performed motion segmentation based on local motion detection based on an optic flow algorithm [4]. The segmentation in this case was implemented by comparing the length of the optic flow vectors with an appropriate threshold.

We employed a simple thresholding for both phase-based motion detection algorithms and optic flow based motion detection. More sophisticated algorithms may produce better segmentation results. Thus, the segmentation was purely based on motion cues and no postprocessing at pixel level was performed. The state-of-the-art results for the Change Detection 2014 dataset utilize multiple cues such as motion, color, and background extraction to segment objects and thereby achieve better results. We are exploring here, however, only the case where motion is the only cue for segmentation. Therefore, the ground truth information from the dataset was not applicable to our test. We will, therefore, only show the effectiveness of motion segmentation visually.

**Figure 16:**
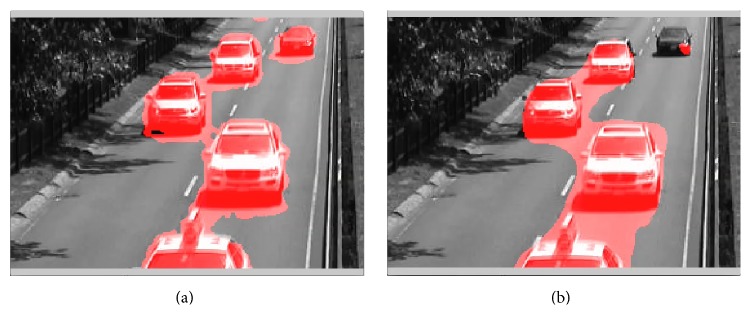
Segmentation of moving cars based on detected motion in the “highway video.” (a) Motion detected by the phase-based motion detection algorithm. (b) Motion detected using optic flow algorithm [4] (see Supplementary Videos S8a and S8b for full videos).

We first applied motion based segmentation on 2-second segment of the “highway video.” The result using the local phase-based motion detection algorithm is shown in the video of Figure 16(a) (see Supplementary Video S8a for full video) and that using optic flow based motion detection algorithm is shown in the video of Figure 16(b) (see Supplementary Video S8b for full video). Both videos are played back at 1/4 of speed. With a higher threshold, the movement of the leaves was no longer picked up by the phase-based motion detector. Therefore, only the cars were identified as moving objects and they are indicated in red. Although the moving objects were not perfectly segmented on their boundary, they were mostly captured. For the optic flow based segmentation, since the regions of interest are set by thresholding the length of the velocity vector, objects moving at lower speed, for example, the cars on the top, were not always picked up by the segmentation algorithm.

**Figure 17:**
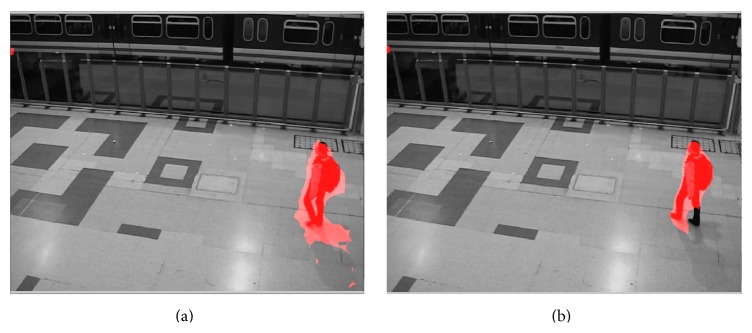
Segmentation of moving people in the “train station video” based on detected motion. (a) Motion detected by the phase-based motion detection algorithm. (b) Motion detected using optic flow algorithm [4] (see Supplementary Videos S9a and S9b for full videos).

**Figure 18:**
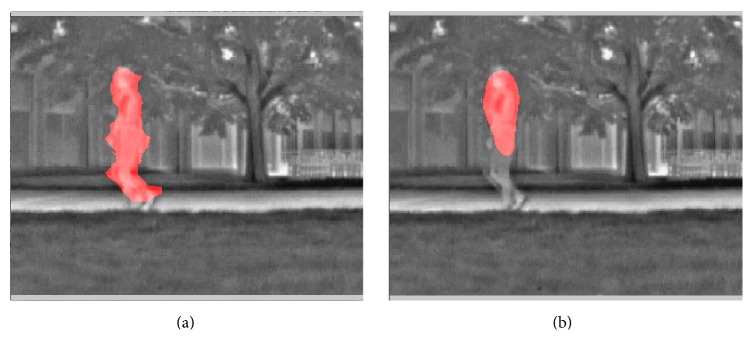
Segmentation of moving people in the “thermal video” based on detected motion. (a) Motion detected by the phase-based motion detection algorithm. (b) Motion detected using optic flow algorithm [4] (see Supplementary Videos S10a and S10b for full videos).

**Figure 19:**
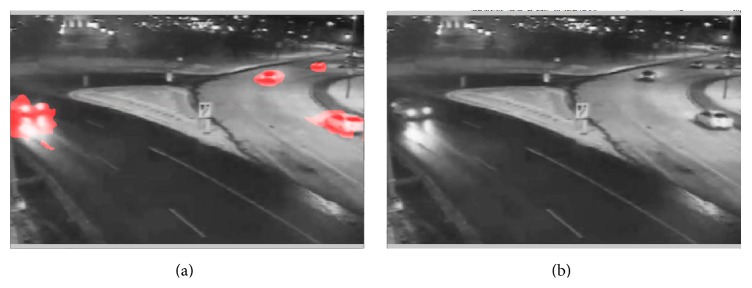
Segmentation of moving cars in the “winter street video” based on detected motion. (a) Motion detected by the phase-based motion detection algorithm. (b) Motion detected using optic flow algorithm [4] (see Supplementary Videos S11a and S11b for full videos).

We then applied the motion segmentation to a 2-second segment of the “train station video,” the “thermal video,” and the “winterstreet video.” The results are shown, respectively, in the videos of Figures 17, 18, and 19 (see Supplementary Videos S9a and S9b, S10a and S10b, and S11a and S11b, resp., for full videos).

These results show that for the purpose of detecting local motion and its use as a motion segmentation cue, the local phase-based motion detector works as good, if not better than a simple thresholding segmentation using an optic flow based algorithm.

## 5. Discussion

Previous research demonstrated that* global* phase information alone can be used to faithfully represent visual scenes. Here we provided a reconstruction algorithm of visual scenes by only using* local* phase information. More importantly, local phase information can be effectively used to detect local motion. Through a simple temporal derivative of the phase, we obtained a second order Volterra kernel that is applied on two normalized inputs. The structure of the second order Volterra kernel in the phase-based motion detector is akin to models employed to detect motion in biological vision, for example, the Reichardt detector [7] and the motion energy detector [9].

We then proposed an efficient, FFT-based algorithm employing the change in local phase for detecting motion. In order to exploit the special structure of the change in phase in the frequency domain that is due to rigid motion, the phase-based motion detection algorithm also incorporates the Radon transform, a transform closely related to the Fourier transform. Based on the Radon transform, a motion indicator was proposed to robustly detect whether the phase change is caused by motion. Therefore, the algorithm can be efficiently implemented whenever the FFT is available/supported. We showed examples of applying the phase-based motion detection algorithm on several video sequences. We also showed that the locally detected motion can be used for segmenting moving objects in video scenes. We compared the segmentation of moving objects using our local phase-based algorithm to segmentation achieved using a widely used optic flow based algorithm. Our results suggest that spatial phase information may provide an efficient alternative to perform many visual tasks* in silico* as well as in modeling* in vivo* biological vision systems. This is consistent with other recent findings [15].

Note that phase information has been used for solving various visual tasks in the past. In fact, phase has been successfully employed in optic flow algorithms [24] and image registration for translation [25], both applied to motion related tasks. The phase-based optic flow algorithm applies the optic flow equation on the local phase of images rather than the intensity itself to achieve better resolution and robustness in estimating motion velocity. The phase correlation method computes the normalized cross-power spectrum of two images to extract the phase difference. It provides better accuracy and robustness as compared to the classical cross correlation approach applied to two consecutive images in a sequence. However, it has limitations when dealing with images with a repetitive structure.

Our method differs from the above two cases in the following ways. First, it employs a simple temporal derivative/high-pass filtering on the phase to extract local phase changes. The structure of the* phase change* is exploited for better detection of motion. On the contrary, the phase correlation method considers the structure of the* phase itself*. This also allowed us to make the motion detection a more local, continuous process rather than purely operating globally on discrete frames. Second, it explores the structure of the change of phase due to motion in the frequency domain rather than in the spatial domain, in which the key constraints of optic flow equations are based. Third, instead of focusing on estimating the exact velocity or shift, our method is centered on the detection of motion with a coarse local estimate of direction. This is the case in the first steps of biological motion detection in the retina or optic lobe of insects; we discussed the resemblance of the method presented here to those of biological models of motion detection.

The proposed motion detection algorithm, however, shares several advantages with the other phase-based methods. For example, it is sensitive to motion that only induces a subpixel shift between frames and for very small differences in intensity. In addition, when compared to the amplitude, the local phase is robust under different contrast and illumination conditions. Consequently, the algorithm presented here can operate in a wide range of contrast/illumination conditions.

Furthermore, once the local phase is extracted from each block, motion detection becomes a localized, temporal operation on the local phase of each block. This forms the basis for the highly parallel structure in the phase-based motion detection algorithm. By contrast, traditional optic flow techniques often rely on explicit comparisons across spatial locations, which increase, for example, memory complexity since all states must be made available to their neighbors.

We also notice that, for each block, a large number of measurements of phase changes are obtained. From Figure 6, we see that this number is much higher than that of the original pixel space. In other words, in order to detect motion in a robust way, the local phase-based motion detection algorithm undergoes an expansion of measurements before settling down onto a single motion indicator value. This number of measurements, however, does not incur additional computational demand thanks to the highly efficient FFT algorithm. Biological visual systems often have a similar structure. For example, in the vertebrate retina, computations are carried out by an extraordinarily large number of neurons until measurements of the visual scene are projected by a fraction of the neurons onto the cortex [26]. Similar expansion takes place in the primary visual cortex as well.

We also highlight the ease of implementation of the intrinsically parallel algorithm proposed here. The algorithm introduced in Section 3.3 is based on the FFT algorithm and does not require solving an optimization problem. It can be efficiently implemented in hardware, for example, FPGAs, or in software, for example, using GPU accelerators. We note that extending the FFT to higher dimensions is straightforward and the implementations of higher dimensional FFTs are also highly efficient. Clearly, the methodology can be applied to motion detection of data in 3D or higher dimensional space, where the Radon transform operates over planes or hyperplanes.

Finally, we argue that the change of phase, although highly nonlinear, can be obtained through a normalization (gain control) followed by a second order Volterra kernel. This separation of a higher order nonlinearity into gain control block and a lower order nonlinear filter can be used for modeling motion detection circuits in biological systems.

## Supplementary Material

Video S1: Video illustrating the evaluation of the Phase Motion Indicator (PMI) for the high-way video sequence. (See Figure 6 in article for descriptions of the subpanels).
Video S2: Full video for Figure 9. Motion detection of the “highway video” using the phase-based algorithm (left), the Reichardt motion detector (middle), and the Barlow-Levick motion detector (right).
Video S3: Full video for Figure 11. Motion detection of the “train station video” using the phase-based algorithm (left), the Reichardt motion detector (middle), and the Barlow-Levick motion detector (right).
Video S4: Full video for Figure 12. Motion detection of the “thermal video” using the phase-based algorithm (left), the Reichardt motion detector (middle), and the Barlow-Levick motion detector (right).
Video S5: Full video for Figure 13. Motion detection of the “winterstreet video” using the phase-based algorithm (left), the Reichardt motion detector (middel), and the Barlow-Levick motion detector (right).
Video S6: Full video for Figure 14. Phase-based motion detection applied on the “highway video” with added Gaussian white noise.
Video S7: Full video for Figure 15. Phase-based motion detection applied on the “train sta-tion video” with added Gaussian white noise.
Video S8a: Full video for Figure 16a. Segmentation of moving cars based on detected mo-tion in the “highway video” using the phase-based motion detection algorithm.
Video S8b: Full video for Figure 16b. Segmentation of moving cars based on detected mo-tion in the “highway video” using optic flow algorithm.
Video S9a: Full video for Figure 17a. Segmentation of moving cars based on detected mo-tion in the “highway video” using the phase-based motion detection algorithm.
Video S9b: Full video for Figure 17b. Segmentation of moving cars based on detected mo-tion in the “train station video” using optic flow algorithm.
Video S10a: Full video for Figure 18a. Segmentation of moving cars based on detected mo-tion in the “thermal video” using the phase-based motion detection algorithm.
Video S10b: Full video for Figure 18b. Segmentation of moving cars based on detected mo-tion in the “thermal video” using optic flow algorithm.
Video S11a: Full video for Figure 19a. Segmentation of moving cars based on detected mo-tion in the “winterstreet video” using the phase-based motion detection algorithm.
Video S11b: Full video for Figure 19b. Segmentation of moving cars based on detected mo-tion in the “winterstreet video” using optic flow algorithm.

## Figures and Tables

**Algorithm 1:**
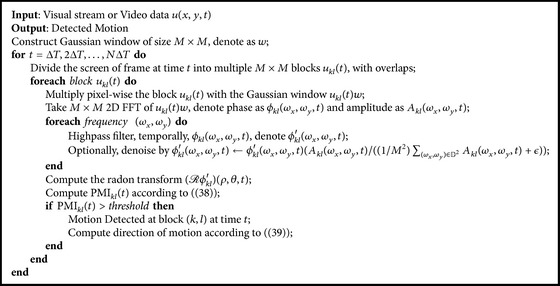
Phase-based motion detection algorithm using the FFT.

**Table 1:** Processing Speeds of the proposed algorithm, the Reichardt motion detector, and the Barlow-Levick Motion detector.

Screen size	Processing capability (frames per second)		
	Proposed	Reichardt	Barlow-Levick
320 × 240	420	790	800
720 × 576	135	685	690
1280 × 720	60	274	293
1920 × 1080	27	191	194

## Appendix

### The Derivative of the Local Phase

Let

(A.1) aωx,ωy,t=∫R2ux,y,twx,ycos⁡ωxx+ωyydx dy,bωx,ωy,t=−∫R2ux,y,twx,ysin⁡ωxx+ωyydx dy,

and, therefore,

(A.2) A00ωx,ωy,tejϕ00ωx,ωy,t=aωx,ωy,t+jbωx,ωy,t.

The local phase amounts to *ϕ*_00_(*ω*_*x*_, *ω*_*y*_, *t*) = arctan⁡2(*b*(*ω*_*x*_, *ω*_*y*_, *t*), *a*(*ω*_*x*_, *ω*_*y*_, *t*)) and therefore

(A.3) $$\begin{array}{c} \frac{d\phi_{00}}{dt}=\frac{a\left(db/dt\right)-b\left(da/dt\right)}{{\left[a\right]}^{2}+{\left[b\right]}^{2}}. \end{array}$$

Now,

(A.4) dadtωx,ωy,t=∫R2∂u∂tx,y,twx,ycos⁡ωxx+ωyydx dy.

For a purely translational signal with instantaneous spatial shift given by (*s*_*x*_(*t*), *s*_*y*_(*t*)) within the support of the window we have

(A.5) dadtωx,ωy,t=−∫R2dsxdtt∂u∂xx,y,twx,ycos⁡ωxx+ωyydx dy−∫R2dsydtt∂u∂yx,y,twx,ycos⁡ωxx+ωyydx dy=dsxdt∫R2ux,y,t∂w∂xx,ycos⁡ωxx+ωyydx dy−ωxdsxdt∫R2ux,y,twx,ysin⁡ωxx+ωyydx dy+dsydt∫R2ux,y,t∂w∂yx,ycos⁡ωxx+ωyydx dy−ωydsydt∫R2ux,y,twx,ysin⁡ωxx+ωyydx dy=dsxdt∫R2ux,y,t∂w∂xx,ycos⁡ωxx+ωyydx dy︸r3=r3ωx,ωy,t+ωxdsxdtb+dsydt∫R2ux,y,t∂w∂yx,ycos⁡ωxx+ωyydx dy︸r4=r4ωx,ωy,t+ωydsydtb.

Similarly,

(A.6) dbdtωx,ωy,t=−dsxdt·∫R2ux,y,t∂w∂xx,ysin⁡ωxx+ωyydx dy︸r5=r5ωx,ωy,t−ωxdsxdta−dsydt·∫R2ux,y,t∂w∂yx,ysin⁡ωxx+ωyydx dy︸r6=r6ωx,ωy,t−ωydsydta.

Plugging in the value of *da*/*dt* and *db*/*dt*, the derivative of the local phase amounts to

(A.7) dϕ00dtωx,ωy,t−dsx/dtr3b+r5a+ωxa2+ωxb2−dsy/dtr4b+r6a+ωya2+ωyb2a2+b2=−dsxdtωx−dsydtωy−dsxdt·r3b+r5aa2+b2−dsydt·r4b+r6aa2+b2︸v00ωx,ωy,t.
